# Aluminum dust exposure and risk of neurodegenerative diseases in a cohort of male miners in Ontario, Canada

**DOI:** 10.5271/sjweh.3974

**Published:** 2021-09-30

**Authors:** Xiaoke Zeng, Jill MacLeod, Colin Berriault, Nathan L DeBono, Victoria H Arrandale, Anne M Harris, Paul A Demers

**Affiliations:** Occupational Cancer Research Centre, Ontario Health, Toronto, ON, Canada; Dalla Lana School of Public Health, University of Toronto, Toronto, ON, Canada; School of Occupational and Public Health, Ryerson University, Toronto, ON, Canada

**Keywords:** Alzheimer’s, epidemiology, metal, parkinsonism, Parkinson’s disease, workplace exposure

## Abstract

**Objectives:**

We estimated associations between respirable aluminum exposure through McIntyre Powder (MP), a fine-sized aluminum and aluminum compound powder, and neurological disease in a retrospective cohort of mining workers from Ontario, Canada. Outcomes included Alzheimer’s disease, Alzheimer’s with other dementias, Parkinson’s disease, parkinsonism, and motor neuron disease.

**Methods:**

The cohort was created by linking a database of mining workers’ work history to healthcare records. This analysis included 36 826 male miners potentially exposed to MP between 1943 and 1979, followed up for disease diagnosis between 1992 and 2018. Exposure was assessed using two approaches, self-reported and historical records. Neurological diseases were ascertained using physician billing and hospital discharge records. Poisson regression models were used to estimate associations between MP exposure and neurological outcomes using incidence rate ratios (RR) and 95% confidence intervals (95% CI).

**Results:**

Exposure to self-reported MP was associated with an elevated incidence rate of Parkinson’s disease (RR 1.34, 95% CI 1.14–1.57). The rate of Parkinson’s disease appeared to increase with the duration of exposure assessed by historical records. Having ever been exposed to MP was positively associated with an elevated rate of Alzheimer’s with other dementias (RR 1.12, 95% CI 1.06–1.19) but not Alzheimer’s disease alone.

**Conclusion:**

This study found that miners who were exposed to MP (respirable aluminum) had elevated rates of Parkinson’s disease. The rate of Parkinson’s disease appeared to increase with the duration of exposure assessed by historical records.

During the mid-to-late 20^th^ century, a purported prophylaxis against silicosis was administered to workers in underground mines through inhalation of McIntyre Powder (MP), an aluminum powder formulation (1). MP prophylaxis was used at various mine sites in Canada, the United States, Mexico, Chile, Australia, and what is today the Democratic Republic of Congo (1). Silicosis is a lung disease caused by exposure to crystalline silica dust that was common among miners at the time (1).

MP was dispersed into an enclosed environment (often the change area or “drys”) exposing workers to short-term and fine-sized insoluble aluminum and aluminum compounds at high concentrations (2). MP was comprised primarily of aluminum oxide (~90%) with metallic aluminum (~10%) (3). A recent study characterized MP, assuming it was produced after 1956, and found that its particles were extremely small – within the nanometer range of 5–100 nm (4). However, MP particles can aggregate to form larger sizes after air suspension. Dispersed MP particles in the air were measured in the range of <200 nanometers to >5 micrometers (3, 5). Before shifts, workers had prescribed exposure to MP with a target concentration of 35.6 milligrams per cubic meter (mg/m^3^) for 10–20 minutes (4). At 15 minutes, this exceeds the threshold limit value for an 8-hour time-weighted average proposed by the American Conference of Governmental Industrial Hygienists (6) and adopted as regulation in many North American jurisdictions. Use of MP was discontinued in the 1970s because of concerns regarding adverse health effects.

The effects of dietary, environmental and occupational exposure to aluminum have been previously examined, primarily focused on Alzheimer’s disease. Findings integrated from eight studies showed an increased risk of Alzheimer’s disease associated with chronic exposure to aluminum from dietary and occupational sources [odds ratio (OR) 1.71 (95% confidence interval (CI) 1.35–2.18)] (7). However, findings between aluminum exposure and the etiology of Alzheimer’s disease remain inconsistent (8), and little evidence has been provided from occupational studies (9–11).

Effects of occupational aluminum exposure on other neurological outcomes have been less studied. Declined cognitive performance has been observed among welders and foundry workers with occupational exposure to aluminum dust and fumes (12–15). Workplace exposure to aluminum was not associated with an increased risk of Parkinson’s disease (16). No published studies were identified investigating associations between occupational aluminum exposure and motor neuron disease. There is limited evidence on the risk of neurological disease due to MP exposure specifically. To date, there have only been two published epidemiological studies: one in Canada (17) and the other in Australia (18), both with small numbers of neurological disease cases.

Using a cohort of underground miners in Ontario, Canada, the objective of this study was to estimate incidence rate ratios (RR) of multiple neurodegenerative diseases (1992–2018), including Alzheimer’s disease (alone and with other dementias), Parkinson’s disease, parkinsonism, and motor neuron disease, among workers exposed to aluminum through MP (1943–1979).

## Methods

### Study design and population

The study cohort was identified using records from the Mining Master File (MMF). The MMF database of 93 526 Ontario underground miners was collected during mandatory annual medical exams held from 1928 to 1988 and includes work history information spanning 1877–1988. These exams certified miners for medical fitness to work underground.

The study cohort included 36 826 male miners in the MMF who had information on name, date of birth, sex, and work history. Miners were eligible if their age at first employment in mining was 15–65 years, age at the start of disease follow-up (1 January 1992) was <100 years, and they were successfully linked to Ontario’s Registered Person’s Database (RPDB). The RPDB contains information on Ontario healthcare recipients with unique identification numbers (health insurance numbers) that enable data linkage to the administrative health service databases. The majority of unlinked miners died or were lost to follow-up (supplementary material www.sjweh.fi/article/3974, figure S1). None of the female mine industry workers linked to RPDB (N=116) were exposed to MP. Women were therefore excluded from further analysis.

### Case ascertainment

Miners who were uniquely identified from the RPDB were then linked to Ontario administrative health databases to identify cases of neurological disease. These databases include the Ontario Health Insurance Plan (OHIP) claims database, which contains health service billing information submitted by healthcare providers; the Discharge Abstract Database (DAD), which contains clinical and demographic information related to hospital discharge; and the National Ambulatory Care Reporting System (NACRS), which contains ambulatory care visits from emergency departments, day surgery, outpatient, and community-based clinics under Canada’s single-payer healthcare system (19). Disease diagnoses in the OHIP were coded with a modified version of ICD-9 (International Classification of Disease, 9^th^ revision) and in DAD and NACRS were coded using ICD-9 before 2002 and ICD-10-CA (Canadian modification of ICD-10) in 2002 and onwards.

Alzheimer’s, Parkinson’s, and motor neuron disease cases were ascertained using hospital discharge or ambulatory care data (at least one record with corresponding diagnostic codes; Alzheimer’s disease: ICD-9 331.0, ICD-10-CA G30; Parkinson’s disease: ICD-9 332.0, ICD-10-CA G20; Motor neuron disease: ICD-9 335.2, ICD-10-CA G12.2). Broader definitions of Alzheimer’s with other dementias and parkinsonism were ascertained using physician billing claims (at least two physician claims with diagnostic codes in OHIP within 12 months; Alzheimer’s with other dementias: 290, 331; Parkinsonism: 332) in addition to at least one record with corresponding diagnostic codes in hospital discharge or ambulatory care data (Alzheimer’s with other dementias: ICD-9 290, 294.1, 331.0, 331.1, 331.82, ICD-10 F00, F01, F02, F03, G30; Parkinsonism: ICD-9 332.0 332.1, ICD-10 G20, G21.0-0.4, G21.8-9, G22, F023) (supplementary table S0). Physician claims data could not be used to isolate Alzheimer’s, Parkinson’s, and motor neuron disease cases because diagnostic codes include these diseases with related conditions (19).

In previous validation studies that investigated various ascertainment algorithms of these health outcomes using Ontario administrative databases, case definitions for Alzheimer’s with other dementias and parkinsonism used in the present study achieved approximately 70% in both positive predictive value and sensitivity (20, 21). Case ascertainment of neurological conditions that only used hospital data also previously showed good positive predictive values. For example, a systematic review reported 56–90% in positive predictive value estimates for Parkinson’s disease from seven studies using hospital data alone with datasets collected in United States, Columbia, and European and Nordic countries (22).

### Exposure assessment

MP was used in many Ontario mines between 1943 and 1979. Miners’ exposure to MP was assessed in this study using two approaches. The first approach used existing self-reported MP exposure information (yes/no) from the MMF database, with data corrected by the research team where available historical records from the McIntyre Research Foundation showed that MP was not administered at a mine in a given year. Miners’ self-reported MP information was collected during the annual medical examination (1951–1979) with prior years (1943–1951) backfilled by the database administrators (ie, Workplace Safety & Insurance Board) using an MP usage list containing 39 gold and 9 uranium mines.

The second approach assigned MP exposure (yes/no) to individuals based on historical records of their working years, mine site, and broad job classification. A comprehensive list of 51 mine sites where MP was administered, including the time period of use, was created using McIntyre Research Foundation records held by the Provincial Archives of Ontario and the historical Mines Accident Prevention Association (MAPAO) dust survey records from the Ontario Ministry of Labor. It was cross-checked with a list reported by the MP Project, a miners’ advocacy organization (23).

MP exposure was categorized into exposed or unexposed for each year of work history using both assessment approaches. MP exposure duration was based on the number of work years with recorded exposure and categorized into >0–1, >1–5, >5–10, and >10 years.

### Cohort set-up and statistical analysis

Miners enrolled in the MMF between 1928 and 1988 were followed up for neurodegenerative disease between 1992 and 2018 (supplementary figure S2). Follow-up started on 1 January 1992 (based on data availability) and ended on the earliest of: diagnosis date for each neurological disease, death or last administrative date of contact with the Ontario health system, or end of the study period on 31 March 2018. Follow-up was censored at age 100 years to reduce potential immortal person-time bias due to loss of follow-up.

All MP-related analyses were conducted independently using both self-reported MP and MP from historical records. Exposure was estimated both as “ever” MP exposure and as duration of MP exposure for a series of exposure periods (only before 1956, ever after 1956, and only after 1956 (5)). Complete results tables from both approaches are presented in supplementary tables S1.1–S5.3).

Time since last MP exposure was categorized into 12–19, 20–29, 30–39, 40–49, and 50–75 years, and compared to non-MP exposed workers. Its association with neurodegenerative outcomes was estimated to infer the latency interval between last exposure and disease diagnosis. Time window analysis was conducted to infer the ‘empirical induction period’, referring to the period between exposure and the first detection of disease (24). This analysis considered neurodegenerative cases and person-times only occurring in each of the assigned time windows (12–19, 20–29, 30–39, 40–49, and 50–75 years ago) and examined RR (95% CI) for each disease between ever and never exposure to MP in that time window.

Poisson regression modeling was used to estimate the association between MP exposure and neurodegenerative disease, adjusted for birth year and age throughout the follow-up period to control for potential confounding by differences in age, birth cohort, and calendar year of follow-up between exposed and unexposed groups. The adjustment variables were specified in the model to maximize model fit based on the Akaike Information Criterion (AIC). The p-value for linear trend across durations of MP exposure was also examined using Poisson regression models. The trend analysis for visual inspection of the slope a linear trend in effect estimates across chosen exposure duration categories. Case counts fewer than six were suppressed as per reporting requirements. All analyses were performed using SAS 9.4 (SAS Institute, Cary, NC, USA). The University of Toronto Health Sciences Research Ethics Board approved this study (protocol # 34944).

## Results

Miners in the study cohort had a median year of birth in 1938 (IQR 1927–1949), median first hire in 1963 (IQR 1952–1971) and median duration of employment as an underground miner of 11 years (IQR 6–18) (table 1). Throughout the follow-up period for neurodegenerative disease, the median follow-up time for miners was 23 years, and their median age at the end of follow-up was 73 years old. According to self-reports approximately 26% of miners in the study cohort were exposed to MP, while 38% had MP exposure according to historical records (table 2). Over 90% of exposed miners experienced at least some of their exposure during the post-1956 period when MP particles were thought to be smaller (5). Miners with self-reported MP exposure tended to be older than unexposed miners (median year of birth 1932 verus 1941), had an earlier year of hire (median 1955 versus 1966), and were employed longer in duration (13 versus 10 years) (table 1). Age at the end of follow up was older for self-reported MP-exposed miners (median age 77 versus 72 years). Using the historical records exposure assessment approach, characteristics of employment duration, total follow-up years, and age at the end of follow-up appeared more similar between exposed and unexposed miners.

**Table 1:** Characteristics of workers in the study cohort and workers with ever and never exposure to McIntyre Powder (MP), described by median and interquartile range. [N=number of workers; IQR=interquartile range].

	Study cohort (N=36 826)	MP-exposed		MP-unexposed	
	
		Self-reports (N=9 548)	Historical records (N=13 827)	Self-reports (N=27 278)	Historical records (N=22 999)
				
	Median (IQR)	Median (IQR)	Median (IQR)	Median (IQR)	Median (IQR)
Year of birth	1938 (1927–1949)	1932 (1923–1941)	1937 (1927–1949)	1941 (1929–1951)	1939 (1927–1949)
Year of first hire	1963 (1952–1971)	1955 (1948–1964)	1958 (1951–1970)	1966 (1954–1973)	1965 (1953–1972)
Duration of employment (years)	11 (6–18)	13 (7–23)	10 (6–19)	10 (6–17)	11 (6–18)
Years of follow-up	23 (11–26)	18 (8–26)	21 (10–26)	25 (12–26)	24 (12–26)
Age at end of follow-up (years)	73 (65–81)	77 (68–83)	73 (65–81)	72 (64–80)	73 (65–81)

**Table 2:** Number and proportion of workers exposed to McIntyre Powder (MP) in the study cohort. The study cohort was created by linking a database of mining workers’ work history (Mining Master File) to healthcare records in Ontario, Canada. This cohort included 36 826 male miners.

MP exposure	Self-reports	Historical records
	
	N (%)	N (%)
Ever exposed	9548 (26)	13 827 (38)
Only before 1956	862 (2)	966 (3)
Ever after 1956	8686 (24)	12 861 (35)
Only after 1956	6459 (18)	10 187 (28)
Never exposed	27 278 (74)	22 999 (62)
Cumulative duration (years) of MP exposure		
>0–1	2296 (6)	4507 (12)
>1–5	3833 (10)	4503 (12)
>5–10	1655 (4)	2433 (7)
>10	1764 (5)	2384 (6)

In the study cohort, ever-exposure to MP using the two assessment approaches was associated with 32–34% increased rate of Parkinson’s disease [from self-reports: RR 1.34 (95% CI 1.14–1.57); from historical records: RR 1.32 (95% CI 1.13–1.54)] (tables 3 and 4). Elevated rates of parkinsonism were observed among MP exposed workers. However, additional analysis showed that no elevation of parkinsonism – excluding Parkinson’s disease cases – was observed, indicating that the elevated rate of parkinsonism among MP-exposed miners was driven by Parkinson’s disease cases, although this analysis only captured a small number of cases from the hospital and ambulatory care data (supplementary table S4.4). Exposure to MP was not associated with Alzheimer’s disease but was associated with a 12–14% increased rate of Alzheimer’s with other dementias [from self-reports: RR 1.12 (95% CI 1.06–1.19); from historical records: RR 1.14 (95% CI 1.08–1.21)]. MP exposure was not associated with motor neuron disease.

**Table 3:** Incidence rate ratios (RR) of neurodegenerative disease (1992–2018) by self-reported McIntyre Powder (MP) exposure (periods and duration of exposure and time since last exposure) among male miners in the study cohort. [CI=confidence interval.] a

MP exposure assessment: Self-reports	Alzheimer’s disease		Alzheimer’s with other dementias		Parkinson’s disease		Parkinsonism		Motor neuron disease	
				
	Cases	RR (95% CI)	Cases	RR (95% CI)	Cases	RR (95% CI)	Cases	RR (95% CI)	Cases	RR (95% CI)
MP unexposed	589	1.00 (‥)	3294	1.00 (‥)	393	1.00 (‥)	667	1.00 (‥)	61	1.00 (‥)
MP exposed	278	0.96 (0.83–1.11)	1721	1.12 (1.06–1.19)	251	1.34 (1.14–1.57)	364	1.19 (1.05–1.36)	20	0.82 (0.49–1.37)
Only before 1956	44	0.90 (0.66–1.23)	260	1.11 (0.98–1.26)	34	1.18 (0.83–1.69)	45	1.08 (0.80–1.48)	<6	‥
Ever after 1956	234	0.97 (0.83–1.13)	1461	1.12 (1.05–1.19)	217	1.36 (1.16–1.61)	319	1.21 (1.06–1.38)	20	0.90 (0.54–1.50)
Only after 1956	122	0.91 (0.75–1.11)	854	1.09 (1.01–1.18)	125	1.34 (1.09–1.64)	192	1.16 (0.99–1.37)	16	1.01 (0.58–1.76)
Duration (year) of exposure										
>0–1	39	0.70 (0.51–0.97)	321	1.03 (0.92–1.15)	50	1.36 (1.01–1.82)	73	1.17 (0.92–1.49)	9	1.62 (0.81–3.27)
>1–5	99	0.97 (0.79–1.21)	636	1.14 (1.05–1.24)	90	1.34 (1.07–1.69)	132	1.18 (0.98–1.42)	<6	‥
>5–10	57	1.04 (0.80–1.37)	323	1.13 (1.01–1.27)	48	1.35 (1.00–1.83)	66	1.15 (0.89–1.48)	<6	‥
>10	83	1.06 (0.84–1.34)	441	1.15 (1.04–1.27)	63	1.30 (0.99–1.71)	93	1.27 (1.02–1.58)	<6	‥
P-value for linear trend		0.469		0.002		0.003		0.030		0.760
Time (year) since last exposure										
12–19	6	0.53 (0.24–1.19)	73	1.26 (0.99–1.60)	12	1.40 (0.78–2.52)	29	1.53 (1.05–2.24)	<6	‥
20–29	41	0.96 (0.69–1.32)	266	1.20 (1.06–1.37)	41	1.39 (1.00–1.93)	73	1.32 (1.02–1.67)	<6	‥
30–39	78	0.99 (0.78–1.26)	500	1.17 (1.07–1.29)	76	1.42 (1.11–1.82)	122	1.27 (1.04–1.54)	9	1.07 (0.53–2.17)
40–49	95	0.98 (0.79–1.22)	551	1.11 (1.02–1.22)	88	1.42 (1.12–1.79)	106	1.15 (0.94–1.42)	<6	‥
50–75	59	0.95 (0.72–1.26)	331	0.99 (0.88–1.11)	35	1.01 (0.71–1.44)	35	0.82 (0.58–1.17)	<6	‥

a All estimates are from Poisson regression models adjusted for age, age^2^, and birth year throughout the follow-up period. The P-value for linear trend was derived from Poisson regression models.

**Table 4:** Incidence rate ratios (RR) of neurodegenerative disease (1992-2018) by McIntyre Powder (MP) exposure assessed from historical records (periods and duration of exposure and time since last exposure) among male miners in the study cohort. [CI=95% confidence interval] a

MP exposure assessment: Historical Records	Alzheimer’s disease		Alzheimer’s with other dementias		Parkinson’s disease		Parkinsonism		Motor neuron disease	
				
	Cases	RR (95% CI)	Cases	RR (95% CI)	Cases	RR (95% CI)	Cases	RR (95% CI)	Cases	RR (95% CI)
MP Unexposed	554	1.00 (‥)	3004	1.00 (‥)	360	1.00 (‥)	605	1.00 (‥)	55	1.00 (–)
MP exposed	313	0.95 (0.82–1.09)	2011	1.14 (1.08–1.21)	284	1.32 (1.13–1.54)	426	1.18 (1.04–1.33)	26	0.80 (0.50–1.28)
Only before 1956	54	1.00 (0.75–1.32)	283	1.11 (0.98–1.26)	39	1.22 (0.87–1.71)	57	1.25 (0.94–1.65)	<6	‥
Ever after 1956	259	0.94 (0.81–1.09)	1728	1.15 (1.08–1.22)	245	1.34 (1.14–1.57)	369	1.17 (1.02–1.33)	26	0.88 (0.55–1.40)
Only after 1956	143	0.93 (0.77–1.13)	1028	1.13 (1.05–1.21)	143	1.33 (1.09–1.62)	230	1.14 (0.98–1.33)	21	0.97 (0.58–1.61)
Duration of MP exposure										
>0–1 year	57	0.95 (0.72–1.24)	417	1.18 (1.06–1.30)	52	1.23 (0.92–1.65)	88	1.12 (0.90–1.41)	10	1.16 (0.59–2.29)
>1–5 years	84	0.89 (0.71–1.12)	591	1.14 (1.04–1.24)	80	1.28 (1.01–1.63)	124	1.15 (0.95–1.40)	6	0.58 (0.25–1.35)
>5–10 years	73	0.96 (0.75–1.22)	428	1.07 (0.96–1.18)	65	1.32 (1.02–1.73)	88	1.09 (0.87–1.36)	<6	‥
>10 years	99	1.00 (0.80–1.23)	575	1.18 (1.08–1.30)	87	1.42 (1.12–1.80)	126	1.32 (1.09–1.61)	7	1.05 (0.48–2.34)
The P-value for linear trend		0.995		0.001		0.003		0.007		0.695
Time since last MP exposure										
12–19 years	6	0.49 (0.22–1.10)	79	1.22 (0.97–1.53)	10	1.05 (0.55–1.99)	34	1.51 (1.06–2.15)	<6	‥
20–29 years	37	0.80 (0.57–1.11)	298	1.21 (1.07–1.36)	45	1.38 (1.00–1.89)	80	1.22 (0.96–1.54)	6	0.94 (0.40–2.22)
30–39 years	90	0.99 (0.80–1.24)	600	1.17 (1.07–1.28)	96	1.50 (1.20–1.88)	147	1.23 (1.03–1.48)	13	1.11 (0.61–2.05)
40–49 years	104	0.95 (0.77–1.18)	631	1.14 (1.04–1.24)	87	1.27 (1.01–1.61)	115	1.10 (0.90–1.34)	<6	‥
50–75 years	76	1.05 (0.82–1.34)	403	1.05 (0.95–1.17)	46	1.14 (0.83–1.56)	50	1.01 (0.75–1.36)	<6	‥

a All estimates are from Poisson regression models adjusted for age, age^2^, and birth year throughout the follow-up period. The P-value for linear trend was derived from Poisson regression models.

By exposure periods, the rate among workers with any post-1956 MP formulation exposure showed a stronger association with Parkinson’s disease than exposure only before 1956 (tables 3 and 4). By duration of MP exposure, the rate of Parkinson’s disease was the greatest among miners with over ten years of MP exposure according to historical records, but this duration trend was not observed for self-reported MP exposure, although the rate remained elevated for all durations of exposure according to self-reports.

The excess rate of Parkinson’s disease and Alzheimer’s with other dementias, followed between 1992 and 2018 in the study cohort, continued for several decades since last MP exposure occurred between 1943 and 1979 (tables 3 and 4). The 40% increased rate of Parkinson’s disease persisted across several decades after the termination of self-reported MP exposure (table 3). Using historical records for MP assessment, the increased rate of Parkinson’s disease reached the highest (50% increased rate) 30–39 years after the exposure ended, then it decreased over time (table 4). In a slightly different pattern, the peak elevated incidence rates of Alzheimer’s with other dementias (~20%) was shown in the 12–19-year post-exposure period and was lessened afterward.

Elevated incidence rates of Parkinson’s disease and Alzheimer’s with other dementias were suggested for one or multiple MP exposure time windows (table 5). The most etiologically relevant MP exposure windows were 30–39 years ago for Parkinson’s disease. The increased rate of Alzheimer’s with other dementias appeared to be in similar magnitudes across several exposure time windows ranging from 12–49 years ago, all of which could be considered etiologically relevant.

**Table 5:** Incidence rate ratios (RR) of neurodegenerative disease (1992–2018) for ever exposure to McIntyre Powder (MP) versus never for various exposure time windows among male miners in the study cohort. [CI=confidence interval.] a

Exposure time windows (number of years ago)	Self-reports					Historical records				
	
	MP exposed		MP-Unexposed		RR (95% CI)	MP exposed		MP-Unexposed		RR (95% CI)
			
	Cases	Cumulative Person-years	Cases	Cumulative Person-years		Cases	Cumulative Person-years	Cases	Cumulative Person-years	
Alzheimer’s disease										
12–19	6	21 076	861	665 989	0.53 (0.24–1.20)	6	42 178	861	644 887	0.50 (0.22–1.13)
20–29	47	57 404	820	629 661	0.88 (0.65–1.19)	43	94 952	824	592 113	0.75 (0.55–1.02)
30–39	116	86 794	751	600 271	0.91 (0.74–1.11)	128	126 819	739	560 246	0.90 (0.74–1.09)
40–49	189	670 784	678	619 981	0.97 (0.82–1.14)	209	87 926	658	599 139	0.95 (0.81–1.11)
50–75	167	26 620	700	660445	1.12 (0.94–1.34)	198	37 663	669	649 402	1.05 (0.89–1.23)
Alzheimer’s with other dementias										
12–19	73	20 937	4942	651 839	1.19 (0.94–1.51)	79	42 019	4936	630 757	1.14 (0.91–1.43)
20–29	333	56 608	4682	616 168	1.16 (1.03–1.30)	366	94 041	4649	578 735	1.15 (1.03–1.28)
30–39	803	84 713	4212	588 063	1.16 (1.08–1.26)	919	124 376	4096	548 400	1.15 (1.07–1.23)
40–49	1153	63 933	3862	608 843	1.15 (1.08–1.23)	1331	84 325	3684	588 451	1.17 (1.09–1.24)
50–75	844	24 052	4171	648 724	1.05 (0.97–1.13)	1056	34 403	3959	638 373	1.05 (0.98–1.13)
Parkinson’s disease										
12–19	12	21 060	632	666 309	1.23 (0.69–2.20)	10	42 165	634	645 204	0.92 (0.49–1.73)
20–29	52	57 397	592	629 972	1.25 (0.93–1.68)	55	94 936	589	592 433	1.20 (0.90–1.60)
30–39	128	86 747	516	600 622	1.37 (1.13–1.68)	144	126 731	500	560 638	1.37 (1.13–1.65)
40–49	175	67 015	469	620 354	1.35 (1.13–1.61)	190	87 857	454	599 512	1.26 (1.06–1.50)
50–75	117	26 721	527	660 648	1.28 (1.04–1.59)	148	37 778	496	649 591	1.27 (1.05–1.55)
Parkinsonism										
12–19	29	20 996	1002	662 811	1.42 (0.97–2.06)	34	42 083	997	641 723	1.41 (0.99–2.00)
20–29	99	57 105	932	626 701	1.28 (1.03–1.58)	110	94 573	921	589 234	1.22 (1.00–1.50)
30–39	215	86 091	816	597 715	1.31 (1.13–1.53)	238	125 966	793	557 840	1.22 (1.05–1.42)
40–49	241	66 215	790	617 591	1.21 (1.04–1.40)	269	86 952	762	596 854	1.12 (0.97–1.29)
50–75	126	26 286	905	657 520	1.10 (0.90–1.34)	164	37 199	867	646 608	1.08 (0.90–1.28)
Motor neuron disease										
12–19	< 6	21 080	79	667 708	‥	2	42 182	79	646 606	0.95 (0.22–4.02)
20–29	< 6	57 478	77	631 309	‥	7	95 032	74	593 756	0.89 (0.40–1.98)
30–39	12	86 989	69	601 799	0.94 (0.51–1.77)	17	127 033	64	561 755	1.05 (0.61–1.81)
40–49	9	67 409	72	621 379	0.61 (0.30–1.23)	13	88 264	68	600 524	0.72 (0.40–1.32)
50–75	6	26 993	75	661 795	0.83 (0.34–1.98)	6	38 118	75	650 670	0.56 (0.24–1.34)

a All estimates are from Poisson regression models adjusted for age, age^2^, and birth year throughout the follow-up period.

## Discussion

This study examined the association between respiratory exposure to aluminum (MP) and neurodegenerative outcomes in a cohort of Ontario underground miners. Our findings show that miners who were exposed to MP had an approximately 30% increased rate of Parkinson’s disease and a 20% increased rate of parkinsonism. However, additional analysis revealed that Parkinson’s disease cases drove the observed increased rate of parkinsonism. Miners with exposure to MP also had a slightly >10% increased rate of Alzheimer’s with other dementias, but no elevated rate of Alzheimer’s alone.

This study of nearly 37 000 miners and >9 500 MP-exposed miners is the first to observe an association between MP and Parkinson’s disease. The two previous studies examining MP exposure and neurological disease were based on a small number of cases with low statistical power (17, 18). Rifat and colleagues (17) reported no significant difference in self- or proxy-reported diagnoses in neurological disorders between MP exposed and unexposed underground miners employed between 1955 and 1979 in Ontario. Among 261 exposed workers, there were only one reported diagnosis of probable Alzheimer’s dementia and three diagnoses of Parkinson’s disease. Among the 346 unexposed miners, only one diagnosis of probable Alzheimer’s dementia was reported. However, with adjustment to age, education, immigrant status, employment duration, head injury, blood pressure during the interview, and several interviewer-related factors, this study found an estimated RR of 2.6 for cognitive function impairment among MP exposed miners. Peters and colleagues (18) followed a cohort of 1894 Australian underground gold miners from 1961 to 2009. Compared to miners without MP exposure, their study suggested an elevated risk of Alzheimer’s disease mortality among Australian gold miners who were exposed to MP [hazard ratio 2.79 (95% CI 0.88–8.82)], but this elevation was based on 16 Alzheimer’s disease deaths. In the present study, MP-exposed miners did not have an elevated incidence rate of Alzheimer’s disease, but did have an increased rate of the broader category of Alzheimer’s with other dementias.

Among MP-exposed miners in this study, case ascertainment methods based on different data sources may explain the observed elevated rate of Alzheimer’s with other dementias but not Alzheimer’s disease alone. Case ascertainment for Alzheimer’s with other dementias has higher sensitivity due to the utilization of both in-patient and outpatient datasets, whereas case definition for Alzheimer’s disease only used in-patient data that may include more severe cases. The elevated rate of Alzheimer’s with other dementias may not rule out the possibility of a small excess of less severe Alzheimer’s disease. In general, the majority of dementia cases may be Alzheimer’s disease. For example, among those aged ≥65 years in North America, Alzheimer’s disease accounted for two-thirds of the prevalent cases of dementia (25). However, in the present study, Alzheimer’s with other dementia may not primarily consist of Alzheimer’s disease alone since outpatient records can include many non-Alzheimer’s cases. For example, one of the outpatient diagnostic codes (OHIP diagnostic code 331) cannot effectively differentiate Alzheimer’s disease from other cerebral degenerations. Hence, the elevated rate of Alzheimer’s with other dementias among exposed miners cannot be used to infer an association between MP and Alzheimer’s disease. However, this elevation may imply that aluminum exposure is associated with a small elevated rate of other types of dementia or cerebral degenerations.

The present study suggests a dose–response relationship between MP exposure duration and Parkinson’s disease. The rate of Parkinson’s disease appeared to increase with the duration of exposure assessed by historical records. Previously, Rifat and colleagues (17) reported an increased range of cognitive impairment with increased MP exposure duration. Peters and colleagues (18) found a suggestive elevated risk of Alzheimer’s disease mortality with years of aluminum dust exposure [hazard ratio 1.11 (95% CI 0.99–1.24), per year of exposure].

Elevated rates of Alzheimer’s with other dementias and Parkinson’s disease were observed with brief MP exposure for less than one year in the current study. It is possible that aluminum reached the brain as a result of the short-term but high-intensity aluminum exposure, resulting in neurotoxic effects. Respirable MP nanoparticles have a high chance of crossing the blood-air gas exchange barrier and entering the bloodstream to be further translocated to other organs (26). Nanoparticles in the bloodstream could then enter the brain by disrupting junctions of endothelial cells in the blood-brain barrier and change its permeability (27). Aluminum nanoparticles may also translocate to the brain through the olfactory bulb after deposition in the nasal epithelium (28, 29). Ultrafine or nanoparticles that reach the brain may initiate a series of neuro-inflammatory activities (30). Exposure to nanoparticles of metal oxide, such as aluminum oxide, can also lead to adverse effects of cytotoxicity and genotoxicity (31), which could contribute to neuron loss, a pathological hallmark in neurodegenerative disease.

### Strengths and limitations

This study of Ontario mining workers is the largest to investigate the association between occupational aluminum exposure and the incidence of neurodegenerative outcomes. This study included approximately 37 000 miners, including >9500 miners with self-reported MP exposure. Of miners from the MMF who were eligible for inclusion for the cohort linkage to neurological disease, we successfully followed up 78% for disease risk. Systematically identifying incident cases from Ontario administrative databases allows for more sensitive identification of cases and statistically precise estimates than case identification using death certificates in previous studies (18, 32), and it reduces recall bias related to cases ascertained from self or proxy reports.

Another significant strength of this study is the utilization of two exposure assessment approaches for MP: self-reports and historical records. The use of historical records may correct for self-reports that are subject to information bias and, in this case, identified a greater number of exposed miners (13 800). In our study, these two approaches generated very consistent results in associations with neurodegenerative outcomes. Lastly, an internal study approach that compared MP-exposed to unexposed miners rather than the general population controlled for bias related to healthy worker effect (33).

This study has several limitations. The start of disease follow-up in the present study was in 1992 because of the availability of electronic health records. Exposure to MP ended in 1979, and the MMF enrollment ended in 1988. As a result, we missed disease cases among the cohort that were diagnosed before 1992, which may cause some disease misclassification for both exposed and unexposed miners. Workers with self-reported MP exposure had a closer median total duration of employment than those unexposed workers (13 versus 10 years), but year of first hire tended to occur in earlier years for exposed than unexposed workers (median 1955 versus 1966), indicating that exposed workers likely were employed in earlier calendar periods than the unexposed. Disease follow-up starting in 1992 may have resulted in more missed cases among the exposed miners than those without, leading to differential disease misclassification. Also, this study was not able to trace approximately 51% of miners in the original MMF file (N=93 526) who died (18%) or were lost to follow-up (19%) before the start of disease follow-up in 1992, and those who otherwise failed to link to Ontario’s hospital and outpatient records (11%). Almost two-thirds of these miners were historically exposed to MP.

This study was not able to quantify the level of aluminum exposure. The administration of MP was known to vary in practice between mines and periods, based on our review of archival and other historical records. Quantitative exposure information was not available in the MMF, and it was not possible for us to estimate workers’ personal MP exposure. We were also not able to adjust for potential mining-related confounders such as exposure to arsenic (34), diesel engine exhaust (32), whole-body vibration (35), or head injury (36). However, we do not expect these other mining-related exposures to be associated with the use of MP in Ontario. We were similarly unable to adjust for non-mining related confounders associated with neurological disease development, such as environmental exposures (37) and other genetic and lifestyle factors.

In identifying cases of neurological disease in this study, the ICD codes at 4 or 5 digits were used in the hospital and ambulatory care databases, allowing for more precise identification of cases. However, physician billing records that contain only the 3-digit code did not allow for the differentiation of Parkinson’s disease from parkinsonism, Alzheimer’s disease from other forms of dementia, and motor neuron diseases from central nervous system diseases. We could not use additional cases from physician billing data to examine rare outcomes such as ALS among motor neuron disease. From hospital and ambulatory care data, 18 ALS cases were identified, but only one ALS case was observed among MP exposed miners. However, we may infer the relationship between MP and ALS from its null association with motor neuron disease. It has been reported that approximately 70% of motor neuron disease cases are ALS (38). Previously, studies found no significant differences in elemental aluminum concentrations in serum (39), cervical spinal cord cells (40), toenail (41), and hair (42), between ALS patients and controls.

### Concluding remarks

This study found that miners who were exposed to MP (respirable aluminum) had elevated rates of Parkinson’s disease and the disease category of Alzheimer’s with other dementias. The rate of Parkinson’s disease appeared to increase with the duration of exposure assessed by historical records and was greater for miners exposed after 1956 when the MP formulation was changed to decrease the particle sizes. Future studies should explore other mining hazards that might contribute to the rate of neurodegenerative disease and health risks that may be associated with aluminum exposure in this population.

## Supplementary material

Supplementary material
