# Supplementary material for: Aluminum dust exposure and risk of neurodegenerative diseases in a cohort of male miners in Ontario, Canada
Source: Scand J Work Environ Health. 2021 Sep 30;47(7):531–9. doi: 10.5271/sjweh.3974 (PMC8504163; doi:10.5271/sjweh.3974)
Supplement: Supplementary material [file SJWEH-47-531-S001.pdf]

Supplemental Materials for manuscript titled, "Aluminum Dust Exposure and Risk of Neurodegenerative Diseases in a Cohort of Male Miners in Ontario, Canada"

Contents

Figure S1. Flow diagram for selection of the study cohort. 2

Figure S2. Timeline of Mining Master File, use of McIntyre Powder, and disease follow-up. 3

Table S0. Case definitions and diagnostics codes used to ascertain Alzheimer's disease, Alzheimer’s with other dementias, Parkinson's disease, parkinsonism, and motor neuron disease. 4

Section 1: Alzheimer’s disease 5

S1.1 Risk of Alzheimer’s disease for ever and cumulative exposure to MP comparing to those never exposed to MP by two assessment approaches. 6

S1.2 Risk of Alzheimer’s disease by time since last MP exposure, overall and by oretype, using two assessment approaches. 7

S1.3 Risk for Alzheimer’s disease for ever exposed to MP versus never for various exposure windows. 8

Section 2: Alzheimer’s and other dementias 9

S2.1 Risk of Alzheimer’s and other dementias for ever and cumulative exposure to MP comparing to those never exposed to MP by two assessment approaches. 10

S2.2 Risk of Alzheimer’s and other dementias by time since last MP exposure, overall and by oretype, using two assessment approaches. 11

S2.3 Risk of Alzheimer’s and other dementias for ever exposed to MP versus never for various exposure windows. 12

Section 3: Parkinsonism 13

S3.1 Risk of parkinsonism for ever and cumulative exposure to MP comparing to those never exposed to MP by two assessment approaches. 14

S3.2 Risk of parkinsonism by time since last MP exposure, overall and by oretype, using two assessment approaches. 15

S3.3 Risk of parkinsonism for ever exposed to MP versus never for various exposure windows. 16

Section 4: Parkinson’s disease 17

S4.1 Risk of Parkinson’s disease for ever and cumulative exposure to MP comparing to those never exposed to MP by two assessment approaches. 18

S4.2 Risk of Parkinson’s disease by time since last MP exposure, overall and by oretype, using two assessment approaches. 19

S4.3 Risk of Parkinson’s disease for ever exposed to MP versus never for various exposure windows. 20

S4.4 Risk of Parkinsonism and Other Parkinsonism but not Parkinson's disease for miners with and without exposure to MP, using self-reports and historical records. 21

Section 5: Motor neuron disease 22

S5.1 Risk of motor neuron disease for ever and cumulative exposure to MP comparing to those never exposed to MP by two assessment approaches. 23

S5.3 Risk of motor neuron disease by time since last MP exposure, overall and by oretype, using two assessment approaches. 24

S5.3 Risk for motor neuron disease for ever exposed to MP versus never for various exposure windows. 25

Figure S1. Flow diagram for selection of the study cohort.

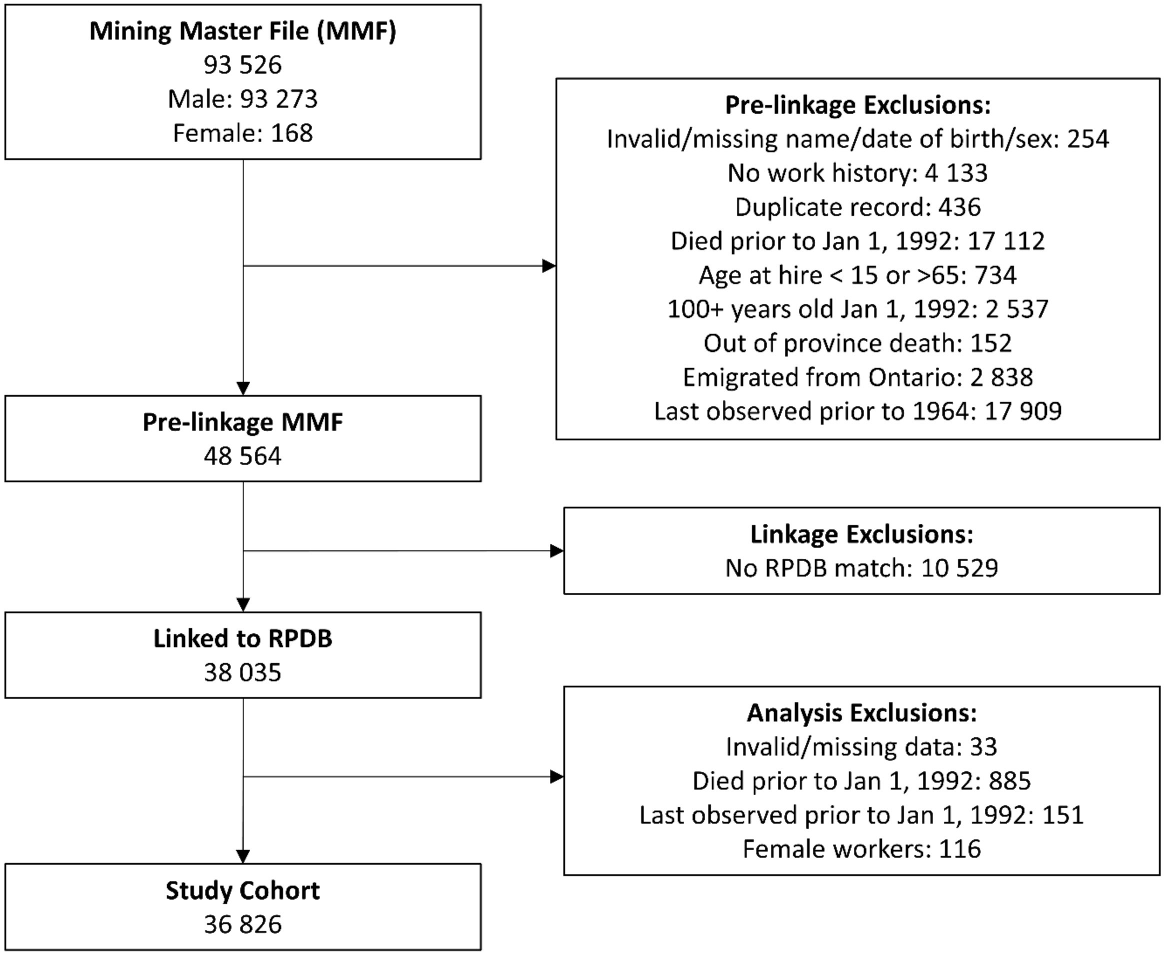

Note: Some records met multiple exclusion criteria; RPDB: Ontario's Registered Persons Database.

Figure S2. Timeline of Mining Master File, use of McIntyre Powder, and disease follow-up.

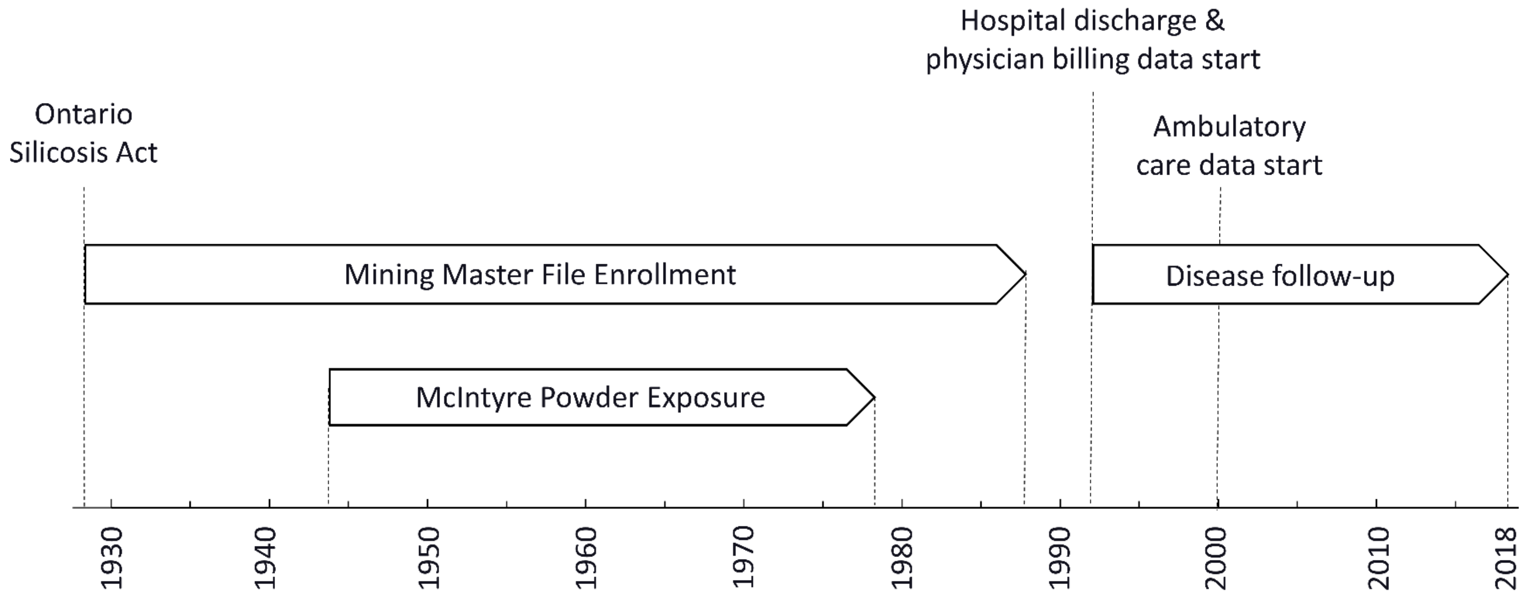

Table S0. Case definitions and diagnostics codes used to ascertain Alzheimer's disease, Alzheimer's with other dementias, Parkinson's disease, parkinsonism, and motor neuron disease.

Neurodegenerative Conditions	DAD/NACRS codes		OHIP codes	Case Definition	Records in DAD	Records in NACRS	Records in OHIP
	ICD-9 (1992-2002)	ICD-10 (2002 onwards)					
Alzheimer's disease	331.0	G30	–	At least one record with corresponding diagnostic codes from hospital discharge or ambulatory care data	≥ 1	≥ 1	–
Alzheimer's with other dementias	290, 294.1, 331.0, 331.1, 331.82	F00, F01, F02, F03, G30	290, 331	At least one record with corresponding diagnostic codes from hospital discharge or ambulatory care data or at least two physician claims with diagnostic codes in OHIP within 12 months	≥ 1	≥ 1	≥ 2 within 12 months
Parkinson's disease	332.0	G20	–	At least one record with corresponding diagnostic codes from hospital discharge or ambulatory care data	≥ 1	≥ 1	–
Parkinsonism	332.0<br>332.1	G20,<br>G21.0-0.4, G21.8-9, G22, F023	332	At least one record with corresponding diagnostic codes from hospital discharge or ambulatory care data or at least two physician claims with diagnostic codes in OHIP within 12 months	≥ 1	≥ 1	≥ 2 within 12 months
Motor neuron disease	335.2	G12.2	–	At least one record with corresponding diagnostic codes from hospital discharge or ambulatory care data	≥ 1	≥ 1	–

ICD: International Classification of Diseases; DAD, Discharge Abstract Database; NACRS, National Ambulatory Care Reporting System; OHIP, Ontario Health Insurance Plan Claims Database; OHIP diagnostic code 290: dementia (senile, presenile); OHIP diagnostic code 331: other cerebral degenerations of central nervous system.

Section 1: Alzheimer’s disease

S1.1 Risk of Alzheimer's disease for ever and cumulative exposure to MP comparing to those never exposed to MP by two assessment approaches.

Exposure metrics			N	Cases	Cumulative person years	RR	LCL	UCL
MP exposure	Self-reports	Never exposed to MP	27 278	589	529 349	1.00	—	—
		Exposed to MP	9 548	278	157 716	0.96	0.83	1.11
		Exposed only before 1956	853	44	8 632	0.90	0.66	1.23
		Exposed ever after 1956	8 695	234	149 084	0.97	0.83	1.13
		Exposed only after 1956	6 459	122	123 399	0.91	0.75	1.11
	Historical Records	Never exposed to MP	22 999	554	438 252	1.00	—	—
		Exposed to MP	13 827	313	248 813	0.95	0.82	1.09
		Exposed only before 1956	966	54	9 457	1.00	0.75	1.32
		Exposed ever after 1956	12 861	259	239 356	0.94	0.81	1.09
		Exposed only after 1956	10 187	143	207 044	0.93	0.77	1.13
Cumulative MP exposure duration	Self-reports (trend P = 0.469)	Never exposed to MP	27 277	589	529 349	1.00	—	—
		> 0 — 1 year	2 296	39	42 017	0.70	0.51	0.97
		> 1 — 5 years	3 833	99	68 117	0.97	0.79	1.21
		> 5 — 10 years	1 655	57	25 690	1.04	0.80	1.37
		> 10 years	1 764	83	21 892	1.06	0.84	1.34
	Historical Records (trend P = 0.995)	Never exposed to MP	22 998	554	438 252	1.00	—	—
		> 0 — 1 year	4 507	57	91 939	0.95	0.72	1.24
		> 1 — 5 years	4 503	84	85 811	0.89	0.71	1.12
		> 5 — 10 years	2 433	73	39 491	0.96	0.75	1.22
		> 10 years	2 384	99	31 572	1.00	0.80	1.23

Alzheimer's disease: 1 code (ICD-9: 331.0; ICD-10: G30) in any diagnostic field in DAD/NACRS; DAD, Discharge Abstract Database; NACRS, National Ambulatory Care Reporting System. MP: McIntyre powder; Self-reports: self-reported MP exposure; Historical Records: estimated MP exposure; N: total number of miners in the analysis group; RR: Incidence rate ratio; LCL: Lower confidence limit; UCL: Upper confidence limit. The trend P value is calculated by fitting a linear line across median values (as continuous) of cumulative MP exposure duration categories in modelling risk of Alzheimer's disease and dementia. All models adjusted by age, age^2, and birth year. Age censored at 100 years old. Reference group: Mining industry workers never exposed to MP.

S1.2 Risk of Alzheimer's disease by time since last MP exposure, overall and by oretype, using two assessment approaches.

Parameter	Time since last exposure	Self-reports					Historical Records				
		Cases	Perуrs	RR	LCL	UCL	Cases	Perуrs	RR	LCL	UCL
Full cohort	Never exposed to MP	589	529 349	1.00	—	—	554	438 252	1.00	—	—
	12–19 years	6	21 076	0.53	0.24	1.19	6	42 178	0.49	0.22	1.10
	20–29 years	41	42 826	0.96	0.69	1.32	37	74 092	0.80	0.57	1.11
	30–39 years	77	54 008	0.99	0.78	1.26	90	83 292	0.99	0.80	1.24
	40–49 years	95	30 172	0.98	0.79	1.22	104	37 188	0.95	0.77	1.18
	50–75 years	59	9 634	0.95	0.72	1.26	76	12 062	1.05	0.82	1.34

Alzheimer's disease: 1 code (ICD-9: 331.0; ICD-10: G30) in any diagnostic field in DAD/NACRS; DAD, Discharge Abstract Database; NACRS, National Ambulatory Care Reporting System. MP: McIntyre powder; Self-reports: self-reported MP exposure; Historical Records: estimated MP exposure; N: total number of miners in the analysis group; Perуrs: cumulative person years; RR: Incidence rate ratio; LCL: Lower confidence limit; UCL: Upper confidence limit. All models adjusted by age, age^2, and birth year. Age censored at 100 years old. *Workers in these groups were employed and exposed to MP only in gold and only in uranium mines; Reference group: Mining industry workers with no MP exposure within the same ore type.

S1.3 Risk for Alzheimer's disease for ever exposed to MP versus never for various exposure windows.

Exposure 'window'	Self-reports							Historical Records						
	MP Exposed		MP-Unexposed		RR	LCL	UCL	MP Exposed		MP-Unexposed		RR	LCL	UCL
	cases	Peryrs	cases	Peryrs				cases	Peryrs	cases	Peryrs			
12–19 years ago	6	21 076	861	665 989	0.53	0.24	1.20	6	42 178	861	644 887	0.50	0.22	1.13
20–29 years ago	47	57 404	820	629 661	0.88	0.65	1.19	43	94 952	824	592 113	0.75	0.55	1.02
30–39 years ago	116	86 794	751	600 271	0.91	0.74	1.11	128	126 819	739	560 246	0.90	0.74	1.09
40–49 years ago	189	67 084	678	619 981	0.97	0.82	1.14	209	87 926	658	599 139	0.95	0.81	1.11
50–75 years ago	167	26 620	700	660 445	1.12	0.94	1.34	198	37 663	669	649 402	1.05	0.89	1.23

Alzheimer's disease: 1 code (ICD-9: 331.0; ICD-10: G30) in any diagnostic field in DAD/NACRS; DAD, Discharge Abstract Database; NACRS, National Ambulatory Care Reporting System. MP: McIntyre powder; Self-reports: self-reported MP exposure; Historical Records: estimated MP exposure; N: total number of miners in the analysis group; Peryrs: cumulative person years; RR: Incidence rate ratio; LCL: Lower confidence limit; UCL: Upper confidence limit. All models adjusted by age, age^2, and birth year. Age censored at 100 years old. Reference group: Mining industry workers with no MP exposure within the same exposure window;

This analysis used a rolling window of exposure and disease follow up. The risk of disease is compared among workers with and without MP exposure in various time windows prior to a given year throughout follow-up. This analysis assumes that those not exposed in each time window as the 'non-exposed'. It also assumes no gaps between first and last exposure dates for all cohort members.

Section 2: Alzheimer's and other dementias

S2.1 Risk of Alzheimer's and other dementias for ever and cumulative exposure to MP comparing to those never exposed to MP by two assessment approaches.

Exposure metrics			N	Cases	Cumulative person years	RR	LCL	UCL
MP exposure	Self-reports	Never exposed to MP	27 273	3294	519 834	1.00	—	—
		Exposed to MP	9 548	1721	152 942	1.12	1.06	1.19
		Exposed only before 1956	862	260	8 096	1.11	0.98	1.26
		Exposed ever after 1956	8 686	1461	144 846	1.12	1.05	1.19
		Exposed only after 1956	6 459	854	120 839	1.09	1.01	1.18
	Historical Records	Never exposed to MP	22 994	3004	429 697	1.00	—	—
		Exposed to MP	13 827	2011	243 079	1.14	1.08	1.21
		Exposed only before 1956	966	283	8 862	1.11	0.98	1.26
		Exposed ever after 1956	12 861	1728	234 216	1.15	1.08	1.22
		Exposed only after 1956	10 187	1028	203 781	1.13	1.05	1.21
Cumulative MP exposure duration	Self-reports (trend P = 0.002)	Never exposed to MP	27 273	3294	519 834	1.00	—	—
		> 0 — 1 year	2 296	321	41 070	1.03	0.92	1.15
		> 1 — 5 years	3 833	636	66 461	1.14	1.05	1.24
		> 5 — 10 years	1 655	323	24 705	1.13	1.01	1.27
		> 10 years	1 764	441	20 705	1.15	1.04	1.27
	Historical Records (trend P = 0.001)	Never exposed to MP	22 994	3004	429 697	1.00	—	—
		> 0 — 1 year	4 507	417	90 698	1.18	1.06	1.30
		> 1 — 5 years	4 503	591	84 114	1.14	1.04	1.24
		> 5 — 10 years	2 433	428	38 242	1.07	0.96	1.18
		> 10 years	2 384	575	30 024	1.18	1.08	1.30

Alzheimer's and other dementias: 1 code (ICD-9: 290, 294.1, 331.0, 331.1, 331.82; ICD-10: G30, F00, F01, F02, F03) in any diagnostic field in DAD/NACRS or 2 physician codes (OHIP code 290 and 331) within 12 months; DAD, Discharge Abstract Database; NACRS, National Ambulatory Care Reporting System; OHIP, Ontario Health Insurance Plan Claims Database. MP: McIntyre powder; Self-reports: self-reported MP exposure; Historical Records: estimated MP exposure; N: total number of miners in the analysis group; RR: Incidence rate ratio; LCL: Lower confidence limit; UCL: Upper confidence limit. The trend P value is calculated by fitting a linear line across median values (as continuous) of cumulative MP exposure duration categories in modelling risk of Alzheimer's disease and dementia. All models adjusted by age, age^2, and birth year. Age censored at 100 years old. Reference group: Mining industry workers never exposed to MP.

S2.2 Risk of Alzheimer's and other dementias by time since last MP exposure, overall and by oretype, using two assessment approaches.

Parameter	Time since last exposure	Self-reports					Historical Records				
		Casess	Peryrs	RR	LCL	UCL	Casess	Peryrs	RR	LCL	UCL
Full cohort	Never exposed to MP	3 294	519 834	1.00	–	–	3 004	429 697	1.00	–	–
	12–19 years	73	20 937	1.26	0.99	1.60	79	42 019	1.22	0.97	1.53
	20–29 years	266	42 161	1.20	1.06	1.37	298	73 318	1.21	1.07	1.36
	30–39 years	500	52 620	1.17	1.07	1.29	600	81 568	1.17	1.07	1.28
	40–49 years	551	28 619	1.11	1.02	1.22	631	35 398	1.14	1.04	1.24
	50–75 years	331	8 605	0.99	0.88	1.11	403	10 776	1.05	0.95	1.17

Alzheimer's and other dementias: 1 code (ICD-9: 290, 294.1, 331.0, 331.1, 331.82; ICD-10: G30, F00, F01, F02, F03) in any diagnostic field in DAD/NACRS or 2 physician codes (OHIP code 290 and 331) within 12 months; DAD, Discharge Abstract Database; NACRS, National Ambulatory Care Reporting System; OHIP, Ontario Health Insurance Plan Claims Database. MP: McIntyre powder; Self-reports: self-reported MP exposure; Historical Records: estimated MP exposure; N: total number of miners in the analysis group; Peryrs: cumulative person years; RR: Incidence rate ratio; LCL: Lower confidence limit; UCL: Upper confidence limit. All models adjusted by age, age^2, and birth year. Age censored at 100 years old. *Workers in these groups were employed and exposed to MP only in gold and only in uranium mines; Reference group: Mining industry workers with no MP exposure within the same ore type.

S2.3 Risk of Alzheimer's and other dementias for ever exposed to MP versus never for various exposure windows.

Exposure 'window'	Self-reports							Historical Records						
	MP Exposed		MP-Unexposed		RR	LCL	UCL	MP Exposed		MP-Unexposed		RR	LCL	UCL
	cases	Peryrs	cases	Peryrs				cases	Peryrs	cases	Peryrs			
12–19 years ago	73	20 937	4 942	651839	1.19	0.94	1.51	79	42 019	4 936	630 757	1.14	0.91	1.43
20–29 years ago	333	56 608	4 682	616168	1.16	1.03	1.30	366	94 041	4 649	578 735	1.15	1.03	1.28
30–39 years ago	803	84 713	4 212	588063	1.16	1.08	1.26	919	124 376	4 096	548 400	1.15	1.07	1.23
40–49 years ago	1 153	63 933	3 862	608843	1.15	1.08	1.23	1331	8 4325	3 684	588 451	1.17	1.09	1.24
50–75 years ago	844	24 052	4 171	648724	1.05	0.97	1.13	1056	34 403	3 959	638 373	1.05	0.98	1.13

Alzheimer's and other dementias: 1 code (ICD-9: 290, 294.1, 331.0, 331.1, 331.82; ICD-10: G30, F00, F01, F02, F03) in any diagnostic field in DAD/NACRS or 2 physician codes (OHIP code 290 and 331) within 12 months; DAD, Discharge Abstract Database; NACRS, National Ambulatory Care Reporting System; OHIP, Ontario Health Insurance Plan Claims Database. MP: McIntyre powder; Self-reports: self-reported MP exposure; Historical Records: estimated MP exposure; N: total number of miners in the analysis group; Peryrs: cumulative person years; RR: Incidence rate ratio; LCL: Lower confidence limit; UCL: Upper confidence limit. All models adjusted by age, age^2, and birth year. Age censored at 100 years old. Reference group: Mining industry workers with no MP exposure within the same exposure window;

This analysis used a rolling window of exposure and disease follow up. The risk of disease is compared among workers with and without MP exposure in various time windows prior to a given year throughout follow-up. This analysis assumes that those not exposed in each time window as the 'non-exposed'. It also assumes no gaps between first and last exposure dates for all cohort members.

Section 3: Parkinsonism

S3.1 Risk of parkinsonism for ever and cumulative exposure to MP comparing to those never exposed to MP by two assessment approaches.

Exposure metrics			N	Cases	Cumulative person years	RR	LCL	UCL
MP exposure	Self-reports	Never exposed to MP	27 274	667	527 379	1.00	—	—
		Exposed to MP	9 547	364	156 428	1.19	1.05	1.36
		Exposed only before 1956	852	45	8 536	1.08	0.80	1.48
		Exposed ever after 1956	8 695	319	147 892	1.21	1.06	1.38
		Exposed only after 1956	6 459	192	122 565	1.16	0.99	1.37
	Historical Records	Never exposed to MP	22 995	605	436 469	1.00	—	—
		Exposed to MP	13 826	426	247 338	1.18	1.04	1.33
		Exposed only before 1956	965	57	9 342	1.25	0.94	1.65
		Exposed ever after 1956	12 861	369	237 996	1.17	1.02	1.33
		Exposed only after 1956	10 187	230	206 087	1.14	0.98	1.33
Cumulative MP exposure duration	Self-reports (trend P = 0.030)	Never exposed to MP	27 278	667	527 379	1.00	—	—
		> 0 — 1 year	2 296	73	41 706	1.17	0.92	1.49
		> 1 — 5 years	3 833	132	67 566	1.18	0.98	1.42
		> 5 — 10 years	1 655	66	25 513	1.15	0.89	1.48
		> 10 years	1 764	93	21 643	1.27	1.02	1.58
	Historical Records (trend P = 0.007)	Never exposed to MP	22 999	605	436 469	1.00	—	—
		> 0 — 1 year	4 507	88	91 714	1.12	0.90	1.41
		> 1 — 5 years	4 503	124	85 217	1.15	0.95	1.40
		> 5 — 10 years	2 433	88	39 206	1.09	0.87	1.36
		> 10 years	2 384	126	31 201	1.32	1.09	1.61

Parkinsonism: 1 code (ICD-9: 332.0, 332.1; ICD-10: G20, G21.0-0.4, G21.8-9, G22, F023) in any diagnostic field in DAD/NACRS or 2 physician codes (OHIP code 332) within 12 months; DAD, Discharge Abstract Database; NACRS, National Ambulatory Care Reporting System; OHIP, Ontario Health Insurance Plan Claims Database. MP: McIntyre powder; Self-reports: self-reported MP exposure; Historical Records: estimated MP exposure; N: total number of miners in the analysis group; RR: Incidence rate ratio; LCL: Lower confidence limit; UCL: Upper confidence limit. The trend P value is calculated by fitting a linear line across median values (as continuous) of cumulative MP exposure duration categories in modelling risk of Alzheimer's disease and dementia. All models adjusted by age, age^2, and birth year. Age censored at 100 years old. Reference group: Mining industry workers never exposed to MP.

S3.2 Risk of parkinsonism by time since last MP exposure, overall and by oretype, using two assessment approaches.

Parameter	Time since last exposure	Self-reports					Historical Records				
		Casess	Peryrs	RR	LCL	UCL	Casess	Peryrs	RR	LCL	UCL
Full cohort	Never exposed to MP	667	527 379	1.00	–	–	605	436 469	1.00	–	–
	12–19 years	29	20 996	1.53	1.05	2.24	34	42 083	1.51	1.06	2.15
	20–29 years	73	42 597	1.31	1.02	1.67	80	73 798	1.22	0.96	1.54
	30–39 years	123	53 556	1.27	1.04	1.54	147	82 722	1.23	1.03	1.48
	40–49 years	105	29 729	1.15	0.94	1.42	115	36 743	1.10	0.90	1.34
	50–75 years	34	9 550	0.82	0.58	1.17	50	11 992	1.01	0.75	1.36

Parkinsonism: 1 code (ICD-9: 332.0, 332.1; ICD-10: G20, G21.0-0.4, G21.8-9, G22, F023) in any diagnostic field in DAD/NACRS or 2 physician codes (OHIP code 332) within 12 months; DAD, Discharge Abstract Database; NACRS, National Ambulatory Care Reporting System; OHIP, Ontario Health Insurance Plan Claims Database. MP: McIntyre powder; Self-reports: self-reported MP exposure; Historical Records: estimated MP exposure; N: total number of miners in the analysis group; Perys: cumulative person years; RR: Incidence rate ratio; LCL: Lower confidence limit; UCL: Upper confidence limit. All models adjusted by age, age^2, and birth year. Age censored at 100 years old. *Workers in these groups were employed and exposed to MP only in gold and only in uranium mines; Reference group: Mining industry workers with no MP exposure within the same ore type.

S3.3 Risk of parkinsonism for ever exposed to MP versus never for various exposure windows.

Exposure 'window'	Self-reports							Historical Records						
	MP Exposed		MP-Unexposed		RR	LCL	UCL	MP Exposed		MP-Unexposed		RR	LCL	UCL
	cases	Peryrs	cases	Peryrs				cases	Peryrs	cases	Peryrs			
12–19 years ago	29	20 996	1002	662 811	1.42	0.97	2.06	34	42 083	997	641 723	1.41	0.99	2.00
20–29 years ago	99	57 105	932	626 701	1.28	1.03	1.58	110	94 573	921	589 234	1.22	1.00	1.50
30–39 years ago	215	86 091	816	597 715	1.31	1.13	1.53	238	125 966	793	557 840	1.22	1.05	1.42
40–49 years ago	241	66 215	790	617 591	1.21	1.04	1.40	269	86 952	762	596 854	1.12	0.97	1.29
50–75 years ago	126	26 286	905	657 520	1.10	0.90	1.34	164	37 199	867	646 608	1.08	0.90	1.28

Parkinsonism: 1 code (ICD-9: 332.0, 332.1; ICD-10: G20, G21.0-0.4, G21.8-9, G22, F023) in any diagnostic field in DAD/NACRS or 2 physician codes (OHIP code 332) within 12 months; DAD, Discharge Abstract Database; NACRS, National Ambulatory Care Reporting System; OHIP, Ontario Health Insurance Plan Claims Database. MP: McIntyre powder; Self-reports: self-reported MP exposure; Historical Records: estimated MP exposure; N: total number of miners in the analysis group; Peryrs: cumulative person years; RR: Incidence rate ratio; LCL: Lower confidence limit; UCL: Upper confidence limit. All models adjusted by age, age^2, and birth year. Age censored at 100 years old. Reference group: Mining industry workers with no MP exposure within the same exposure window;

This analysis used a rolling window of exposure and disease follow up. The risk of disease is compared among workers with and without MP exposure in various time windows prior to a given year throughout follow-up. This analysis assumes that those not exposed in each time window as the 'non-exposed'. It also assumes no gaps between first and last exposure dates for all cohort members.

Section 4: Parkinson’s disease

S4.1 Risk of Parkinson's disease for ever and cumulative exposure to MP comparing to those never exposed to MP by two assessment approaches.

Exposure metrics			N	Cases	Cumulative person years	RR	LCL	UCL
MP exposure	Self-reports	Never exposed to MP	27 278	393	529 698	1.00	–	–
		Exposed to MP	9 548	251	157 671	1.34	1.14	1.57
		Exposed only before 1956	853	34	8 634	1.18	0.83	1.69
		Exposed ever after 1956	8 695	217	149 037	1.36	1.16	1.61
		Exposed only after 1956	6 459	125	123 325	1.34	1.09	1.64
	Historical Records	Never exposed to MP	22 999	360	438 584	1.00	–	–
		Exposed to MP	13 827	284	248 785	1.32	1.13	1.54
		Exposed only before 1956	966	39	9 499	1.22	0.87	1.71
		Exposed ever after 1956	12 861	245	239 286	1.34	1.14	1.57
		Exposed only after 1956	10 187	143	206 963	1.33	1.09	1.62
Cumulative MP exposure duration	Self-reports (trend P = 0.043)	Never exposed to MP	27 274	393	529 698	1.00	–	–
		> 0 – 1 year	2 296	50	41 960	1.36	1.01	1.82
		> 1 – 5 years	3 833	90	68 090	1.34	1.07	1.69
		> 5 – 10 years	1 654	48	25 693	1.35	1.00	1.83
		> 10 years	1 764	63	21 928	1.30	0.99	1.71
	Historical Records (trend P = 0.003)	Never exposed to MP	22 995	360	438 584	1.00	–	–
		> 0 – 1 year	4 507	52	91 987	1.23	0.92	1.65
		> 1 – 5 years	4 503	80	85 742	1.28	1.01	1.63
		> 5 – 10 years	2 432	65	39 465	1.32	1.02	1.73
		> 10 years	2 384	87	31 591	1.42	1.12	1.80

Parkinson's disease: 1 code (ICD-9: 332.0; ICD-10: G20) in any diagnostic field in DAD/NACRS; DAD, Discharge Abstract Database; NACRS, National Ambulatory Care Reporting System. MP: McIntyre powder; Self-reports: self-reported MP exposure; Historical Records: estimated MP exposure; N: total number of miners in the analysis group; RR: Incidence rate ratio; LCL: Lower confidence limit; UCL: Upper confidence limit. The trend P value is calculated by fitting a linear line across median values (as continuous) of cumulative MP exposure duration categories in modelling risk of Alzheimer's disease and dementia. All models adjusted by age, age^2, and birth year. Age censored at 100 years old. Reference group: Mining industry workers never exposed to MP.

S4.2 Risk of Parkinson's disease by time since last MP exposure, overall and by oretype, using two assessment approaches.

Parameter	Time since last exposure	Self-reports					Historical Records				
		Casess	Peryrs	RR	LCL	UCL	Casess	Peryrs	RR	LCL	UCL
Full cohort	Never exposed to MP	393	529 698	1.00	—	—	360	438 584	1.00	—	—
	12–19 years	12	21 060	1.40	0.78	2.52	10	42 165	1.05	0.55	1.99
	20–29 years	41	42 830	1.39	1.00	1.93	45	74 090	1.38	1.00	1.89
	30–39 years	77	53 969	1.42	1.11	1.82	96	83 220	1.50	1.20	1.88
	40–49 years	86	30 109	1.42	1.12	1.79	87	37 139	1.27	1.01	1.61
	50–75 years	35	9 703	1.01	0.71	1.45	46	12 171	1.14	0.83	1.56

Parkinson's disease: 1 code (ICD-9: 332.0; ICD-10: G20) in any diagnostic field in DAD/NACRS; DAD, Discharge Abstract Database; NACRS, National Ambulatory Care Reporting System. MP: McIntyre powder; Self-reports: self-reported MP exposure; Historical Records: estimated MP exposure; N: total number of miners in the analysis group; Perys: cumulative person years; RR: Incidence rate ratio; LCL: Lower confidence limit; UCL: Upper confidence limit. All models adjusted by age, age^2, and birth year. Age censored at 100 years old. *Workers in these groups were employed and exposed to MP only in gold and only in uranium mines; Reference group: Mining industry workers with no MP exposure within the same ore type.

S4.3 Risk of Parkinson's disease for ever exposed to MP versus never for various exposure windows.

Exposure 'window'	Self-reports							Historical Records						
	MP Exposed		MP-Unexposed		RR	LCL	UCL	MP Exposed		MP-Unexposed		RR	LCL	UCL
	cases	Peryrs	cases	Peryrs				cases	Peryrs	cases	Peryrs			
12–19 years ago	12	21 060	632	666 309	1.23	0.69	2.20	10	42 165	634	645 204	0.92	0.49	1.73
20–29 years ago	52	57 397	592	629 972	1.25	0.93	1.68	55	94 936	589	592 433	1.20	0.90	1.60
30–39 years ago	128	86 747	516	600 622	1.37	1.13	1.68	144	126 731	500	560 638	1.37	1.13	1.65
40–49 years ago	175	67 015	469	620 354	1.35	1.13	1.61	190	87 857	454	599 512	1.26	1.06	1.50
50–75 years ago	117	26 721	527	660 648	1.28	1.04	1.59	148	37 778	496	649 591	1.27	1.05	1.55

Parkinson's disease: 1 code (ICD-9: 332.0; ICD-10: G20) in any diagnostic field in DAD/NACRS; DAD, Discharge Abstract Database; NACRS, National Ambulatory Care Reporting System. MP: McIntyre powder; Self-reports: self-reported MP exposure; Historical Records: estimated MP exposure; N: total number of miners in the analysis group; Peryrs: cumulative person years; RR: Incidence rate ratio; LCL: Lower confidence limit; UCL: Upper confidence limit. All models adjusted by age, age^2, and birth year. Age censored at 100 years old. Reference group: Mining industry workers with no MP exposure within the same exposure window;

This analysis used a rolling window of exposure and disease follow up. The risk of disease is compared among workers with and without MP exposure in various time windows prior to a given year throughout follow-up. This analysis assumes that those not exposed in each time window as the 'non-exposed'. It also assumes no gaps between first and last exposure dates for all cohort members.

S4.4 Risk of Parkinsonism and Other Parkinsonism but not Parkinson's disease for miners with and without exposure to MP, using self-reports and historical records.

Conditions	Case definition	MP exposure		Cases	peryrs	RR	LCL	UCL
Parkinsonism	1 code (ICD-9 332.0, 332.1; ICD-10 G20, G21.0-0.4, G21.8-9, G22, F02.3) in any diagnostic field in DAD/NACRS	Self-reports	Ever	255	157 443	1.32	1.13	1.54
			Never	406	529 346	1.00	—	—
		Historical records	Ever	289	248 543	1.30	1.11	1.51
			Never	372	438 246	1.00	—	—
Other parkinsonism but not Parkinson's disease	1 code (ICD-9 332.1; ICD-10 G21.0-0.4, G21.8-9, G22, F02.3) in any diagnostic field in DAD/NACRS	Self-reports	Ever	4	158 259	0.75	0.24	2.34
			Never	13	530 616	1.00	—	—
		Historical records	Ever	5	249 450	0.71	0.25	2.01
			Never	12	439 425	1.00	—	—
Other parkinsonism but not comfirmed Parkinson's disease	1 code (ICD-9 332.1; ICD-10 G21.0-0.4, G21.8-9, G22, F02.3) in any diagnostic field in DAD/NACRS or 2 physician codes (OHIP code 332) within 12 months	Self-reports	Ever	360	156 449	1.20	1.06	1.37
			Never	421	527 432	1.00	—	—
		Historical records	Ever	593	247 363	1.19	1.05	1.34
			Never	421	436 518	1.00	—	—

DAD, Discharge Abstract Database; NACRS, National Ambulatory Care Reporting System; OHIP, Ontario Health Insurance Plan Claims Database. MP: McIntyre powder; Self-reports: self-reported MP exposure; Historical Records: estimated MP exposure; N: total number of miners in the analysis group; Peryrs: cumulative person years; RR: Incidence rate ratio; LCL: Lower confidence limit; UCL: Upper confidence limit. All models adjusted by age, age^2, and birth year. Age censored at 100 years old.

Section 5: Motor neuron disease

S5.1 Risk of motor neuron disease for ever and cumulative exposure to MP comparing to those never exposed to MP by two assessment approaches.

Exposure metrics			N	Cases	Cumulative person years	RR	LCL	UCL
MP exposure	Self-reports	Never exposed to MP	27 278	61	530 544	1.00	—	—
		Exposed to MP	9 548	20	158 243	0.82	0.49	1.37
		Exposed only before 1956	853	0	8 718	—	—	—
		Exposed ever after 1956	8 695	20	149 526	0.90	0.54	1.50
		Exposed only after 1956	6 459	16	123 654	1.01	0.58	1.76
	Historical Records	Never exposed to MP	22 999	55	439 363	1.00	—	—
		Exposed to MP	13 827	26	249 425	0.80	0.50	1.28
		Exposed only before 1956	966	0	9 573	—	—	—
		Exposed ever after 1956	12 861	26	239 852	0.88	0.55	1.40
		Exposed only after 1956	10 187	21	207 347	0.97	0.58	1.61
Cumulative MP exposure duration	Self-reports (trend P = 0.760)	Never exposed to MP	27 278	61	530 544	1.00	—	—
		> 0 — 1 year	2 296	9	42 089	1.62	0.81	3.27
		> 1 — 5 years	3 833	<6	68 310	0.31	0.10	1.00
		> 5 — 10 years	1 655	<6	25 805	0.68	0.21	2.16
		> 10 years	1 764	<6	22 040	1.05	0.41	2.68
	Historical Records (trend P = 0.695)	Never exposed to MP	22 999	55	439 363	1.00	—	—
		> 0 — 1 year	4 507	10	92 096	1.16	0.59	2.29
		> 1 — 5 years	4 503	6	85 947	0.58	0.25	1.35
		> 5 — 10 years	2 433	<6	39 617	0.45	0.14	1.44
		> 10 years	2 384	7	31 765	1.05	0.48	2.34

Motor neuron disease: 1 code (ICD-9: 335.2; ICD-10: G12.2) in any diagnostic field in DAD/NACRS; DAD, Discharge Abstract Database; NACRS, National Ambulatory Care Reporting System. MP: McIntyre powder; Self-reports: self-reported MP exposure; Historical Records: estimated MP exposure; N: total number of miners in the analysis group; RR: Incidence rate ratio; LCL: Lower confidence limit; UCL: Upper confidence limit. The trend P value is calculated by fitting a linear line across median values (as continuous) of cumulative MP exposure duration categories in modelling risk of Alzheimer's disease and dementia. All models adjusted by age, age^2, and birth year. Age censored at 100 years old. Reference group: Mining industry workers never exposed to MP.

S5.3 Risk of motor neuron disease by time since last MP exposure, overall and by oretype, using two assessment approaches.

Parameter	Time since last exposure	Self-reports					Historical Records				
		Casess	Peryrs	RR	LCL	UCL	Casess	Peryrs	RR	LCL	UCL
Full cohort	Never exposed to MP	61	530 544	1.00	–	–	55	439 363	1.00	–	–
	12–19 years	< 6	21 080	–	–	–	< 6	42 182	–	–	–
	20–29 years	< 6	42 894	–	–	–	6	74 167	0.94	0.40	2.22
	30–39 years	9	54 134	1.07	0.53	2.17	13	83 435	1.11	0.61	2.05
	40–49 years	< 6	30 340	–	–	–	< 6	37 362	–	–	–
	50–75 years	< 6	9 797	–	–	–	< 6	12 279	–	–	–

Motor neuron disease: 1 code (ICD-9: 335.2; ICD-10: G12.2) in any diagnostic field in DAD/NACRS; DAD, Discharge Abstract Database; NACRS, National Ambulatory Care Reporting System. MP: McIntyre powder; Self-reports: self-reported MP exposure; Historical Records: estimated MP exposure; N: total number of miners in the analysis group; Peryrs: cumulative person years; RR: Incidence rate ratio; LCL: Lower confidence limit; UCL: Upper confidence limit. All models adjusted by age, age^2, and birth year. Age censored at 100 years old. *Workers in these groups were employed and exposed to MP only in gold and only in uranium mines; Reference group: Mining industry workers with no MP exposure within the same ore type.

S5.3 Risk for motor neuron disease for ever exposed to MP versus never for various exposure windows.

Exposure 'window'	Self-reports							Historical Records						
	MP Exposed		MP-Unexposed		RR	LCL	UCL	MP Exposed		MP-Unexposed		RR	LCL	UCL
	cases	Peryrs	cases	Peryrs				cases	Peryrs	cases	Peryrs			
12–19 years ago	2	21 080	79	66 7708	1.33	0.32	5.58	2	42 182	79	646 606	0.95	0.22	4.02
20–29 years ago	4	57 478	77	63 1309	0.66	0.24	1.82	7	95 032	74	593 756	0.89	0.40	1.98
30–39 years ago	12	86 989	69	60 1799	0.95	0.51	1.76	17	127 033	64	561 755	1.05	0.61	1.81
40–49 years ago	9	67 409	72	62 1379	0.61	0.30	1.24	13	88 264	68	600 524	0.72	0.40	1.32
50–75 years ago	6	26 993	75	66 1795	0.83	0.34	1.99	6	38 118	75	650 670	0.56	0.24	1.34

Motor neuron disease: 1 code (ICD-9: 335.2; ICD-10: G12.2) in any diagnostic field in DAD/NACRS; DAD, Discharge Abstract Database; NACRS, National Ambulatory Care Reporting System. MP: McIntyre powder; Self-reports: self-reported MP exposure; Historical Records: estimated MP exposure; N: total number of miners in the analysis group; Peryrs: cumulative person years; RR: Incidence rate ratio; LCL: Lower confidence limit; UCL: Upper confidence limit. All models adjusted by age, age^2, and birth year. Age censored at 100 years old. Reference group: Mining industry workers with no MP exposure within the same exposure window;

This analysis used a rolling window of exposure and disease follow up. The risk of disease is compared among workers with and without MP exposure in various time windows prior to a given year throughout follow-up. This analysis assumes that those not exposed in each time window as the 'non-exposed'. It also assumes no gaps between first and last exposure dates for all cohort members.
